# Value assessment of animal protein can help feed the future

**DOI:** 10.1093/af/vfae006

**Published:** 2024-04-16

**Authors:** Bucky Gwartney

**Affiliations:** Agricultural Marketing Service, USDA, Washington, DC, USA

We have all read the predictions and implications for global population growth and the expectations for producing a food supply to match. I have no doubt that global food producers will be able to meet that demand, but it will not be easy. Certainly, high-quality, nutrient dense proteins will be a major part of that effort. But there are a lot of head winds against increasing food production, weather pattern shifts, water scarcity, reduction of arable land, and reduced labor to name a few, so how is this possible? I do not think it is a secret that the improvement in food production through the adoption of technology that improves crop yields, reduces water usage, and improves animal genetics, is one of the keys to achieve these goals. A key factor in a more sustainable global beef production system is economics and ensuring efficiencies within the supply chain.

So, what does measuring value in livestock and meat have to do with this? Economic signals play a large role, and without them, food production systems would be much slower to change, to adopt technology, and to invest their capital on the equipment needed for improvements. Change is hard, and people need compelling reasons to change. The articles in this edition of *Animal Frontiers* continue the conversation surrounding value determination. Granted, this review is merely a snapshot of the global food production system and focuses mainly on beef with a nod to lamb production. The unique aspects of the ruminant species provide an interesting perspective, mainly due to their lack of consolidation or integration, when compared to other proteins such as poultry or pork. The reliance and utilization of feedstuffs not normally used for other animal production allows for a very diverse set of production systems, breeds of animals, and value propositions around the world. The beauty of these species is that they can be used productively in all parts of the world with fewer requirements and inputs.

An integral part of the value proposition is measuring important production and animal characteristics while being able to apply those measurements against a standard that is not only robust in its criteria but also meaningful in its outcome. While North America is not in this edition, it excels in its utilization of standards, data and market reporting, criteria that build a solid foundation of lifecycle information allowing food production systems to thrive. These mature and robust lifecycle information systems are the largest in the world and are utilized by many third-party users to build risk management systems and communicate value propositions across many commodities. Food security in developing countries is important and a function that governments assist and advise. One of the first learnings when assisting a country in need of food security and increased food production is evaluating its market reporting abilities. The basic collection and reporting systems start the process of communicating the value of their livestock within the food production chain. In many situations, the only thing being measured is the weight of the animal, and while this can relay a value signal in that situation, it is limiting. Remember, utilizing measurements, collecting data, and segregating into value classes does not have to start out as a sophisticated scheme. It can begin as rudimentary measures of animal and carcass dimensions, or breed type classifications, or yield precision based on simple, basic parameters. So, it is critically important to start the process of building a framework for this type of value determination, because without it, growth and differentiation in the product and the system will progress very slowly.

When we think of vast grasslands, and wide-open spaces, we might go to South America in our minds. We curated three countries from South America to introduce to us their work and vision for measuring and creating value in cattle and beef protein. What is interesting to me as I review these articles is not only the commonalities of the regions but also some of the unique differences. For example, in the Uruguay, Brazil, and Chile articles (Arias, 2024; Brito et al., 2024; Nunes et al., 2024), they all highlight the use of a standards type approach for determining a grade category and thus a value. While some of the systems are mandated by the government, others are not. In some cases, these are government mandated programs, but in many cases, stakeholder and market-driven programs that evaluate value can be far superior. So, these three countries typify a lot of what you see in other countries that have a highly varied production system. While the current systems work, there are desires to change them, to implement more quality-oriented parameters, to meet an increased global demand and export requirements. In most cases, these countries do not look at the quality parameters related to the amount of intramuscular fat, a result of feeding high-energy diets to the animal. However, export demand can carve out certain independent sectors of the industry to fill the global demand for high-quality, grain fed beef. The development and adoption of these systems is driven by the need to further classify the existing populations into value groups, and that will continue.

Australia provides a unique perspective on their value assessment systems (Stewart et al., 2024). Not unlike other countries, they utilize standards, and basic measurements to place value differentiation on their meat protein sector, including beef and lamb. But they have also developed a consumer-based system that takes the value proposition to the next level. Being a high export country for their beef and lamb products, value differentiation becomes even more important, to not only meet their own domestic needs, but to fit the various global consumers as well.

The global landscape for animal protein production is changing faster than it ever has, and with the new artificial intelligence (AI)-based tools becoming more prevalent, it is not going to slow down. Speaking of AI, it is going to be a game changer, so the industry must be engaged on how these tools are developed, managed, and applied to our industry.

I hope you enjoy reading these articles from around the world and appreciate the diversity that the global meat-producing industry has in the way it not only produces animals and meat but also the sophistication of the various systems. Having production systems ranging from very basic to sophisticated is the norm, but transitioning those basic systems using technology, standards, measurements, and data will not only transform those basic simple systems into more viable enterprises, they will also result in an increased supply of safe, quality protein for the world’s consumers. Bon Appetit!

## About the Author

**Bucky Gwartney** is currently serving a detail in the Livestock & Poultry Program’s Office of the Deputy Administrator. In that role he is coordinating a number of AMS program initiatives that have a high level of stakeholder awareness and engagement. He also continues to support the Standards and Specifications Division with certain aspects of his role as International Marketing Specialist. He participates in multiple national and international standards bodies and industry associations, representing AMS in leadership roles. He received his PhD in Animal Science at the University of Nebraska and a BS in Agriculture from Oklahoma State University. **Corresponding author:**bucky.gwartney@usda.gov
